# Wide-Band Circularly Polarized ReflectarrayUsing Graphene-Based Pancharatnam-Berry Phase Unit-Cells for Terahertz Communication

**DOI:** 10.3390/ma11060956

**Published:** 2018-06-05

**Authors:** Li Deng, Yuanyuan Zhang, Jianfeng Zhu, Chen Zhang

**Affiliations:** Beijing Key Laboratory of Network System Architecture and Convergence, School of Information and Communication Engineering, Beijing University of Posts and Telecommunications, Beijing 100876, China; zhangyuanyuan@bupt.edu.cn (Y.Z.); zhujianfeng@bupt.edu.cn (J.Z.); zhangchenzc@bupt.edu.cn (C.Z.)

**Keywords:** circularly polarized, Pancharatnam Berry phase, graphene, metasurface

## Abstract

A wide-band and high gain circularly polarized (CP) graphene-based reflectarray operating in the THz regime is proposed and theoretically investigated in this paper. The proposed reflectarray consists of a THz CP source and several graphene-based unit-cells. Taking advantages of the Pancharatnam Berry (PB) phase principle, the graphene-based unit-cell is capable of realizing a tunable phase range of 360° in a wide-band (1.4–1.7 THz) by unit-cell rotating, overcoming the restriction of intrinsic narrow-band resonance in graphene. Therefore, this graphene-based unit-cell exhibits superior bandwidth and phase tunability to its previous counterparts. To demonstrate this, a wide-band (1.4–1.7 THz) focusing metasurface based on the proposed unit-cell that exhibits excellent focusing effect was designed. Then, according to the reversibility of the optical path, a CP reflectarray was realized by placing a wide-band CP THz source at the focal point of the metasurface. Numerical simulation demonstrates that this reflectarray can achieve a stable high gain up to 15 dBic and an axial ratio around 2.1 dB over the 1.4–1.7 THz band. The good radiation performance of the proposed CP reflectarray, as demonstrated, underlines its suitability for the THz communication applications. Moreover, the design principle of this graphene-based reflectarray with a full 360° phase range tunable unit-cells provides a new pathway to design high-performance CP reflectarray in the THz regime.

## 1. Introduction

Terahertz (THz) science and technology that is undergoing an unprecedented rapid development has attracted considerable attention in the scientific community. As a promising technology, it has enormous potential in applications such as communication [1], sensing [2], imaging [3], detection [4] and spectroscopy [5]. In terms of communication applications, the THz communication system is particularly promising as it has a sufficient bandwidth that is capable of handling high data rate up to 100 Gb/s [6]. Consequently, new requirements for THz antennas have been put forward. Generally, high gain THz antennas are indispensable due to the intrinsic high path loss characteristic of THz waves in free space. Besides, considering the performance deterioration of linearly polarized antennas when they are polarization misaligned, circularly polarized (CP) antennas are often required in scenarios where multi-path effect are prominent. Therefore, high gain CP THz antennas are highly desirable.

Reflectarrays [7,8,9,10,11,12], which consist of an array of passive cells, are well suited for high gain THz applications [13,14]. They combine the merits of both conventional reflect and array antennas, namely, low loss, compact profile, low cross polarization, easy manufacturing, and high efficiency. By controlling the phase distribution on the array surface, the far-field radiation properties can be easily manipulated without bulky and lossy beamforming networks. In terms of CP reflectarray, several CP reflectarrays with excellent performances have been designed at microwave or millimeter wave frequencies [15,16,17,18,19]. It seems that reflectarrays can be chosen as a competitive candidate for high gain and CP THz applications. Nevertheless, most of these reflectarrays rely on judiciously designed metallic structures, which cannot simply scale to the THz frequencies because of the prominent skin effect of the conventional metal.The ohm loss of the metallic will immensely affect the array gain and radiation efficiency. Toavoid these drawbacks brought by the skin effect of metals at THz frequencies, there is a great demand for new types of THz antenna. Fortunately, graphene has several advantages over convention metals at the THz band. It has a high electron mobility up to 230,000 cm^2^/Vs at room temperature [20], and a low electrical resistivity about 10^−6^ Ω·cm [21,22] in THz band, demonstrating lower loss than conventional metals. In addition, the surface plasmons resonant frequencies of graphene are quite lower than that in metals, which are often in optical frequencies. Meanwhile, graphene surface plasmons exhibit extremely small wavelengths (*λ*/10–*λ*/100) and tight field confinement on the graphene sheet [23,24], while maintaining reasonably small losses in the THz band. Furthermore, the imaginary conductivity of graphene is highly tunable via chemical doping or electrical gating [25,26,27,28,29,30,31] at THz frequencies, which is impossible or inefficient if metals are used. Based on excellent physical properties of graphene at THz frequencies, several graphene-based THz antennas have been reported in recent years [32,33,34,35,36,37,38,39,40,41]. It evaluates the feasibility of a fixed beam reflectarray antenna at THz based on graphene and compares its performance to a similar implementation using gold for the first time [42]. Soon after, diverse graphene-based reflectarrays operating at THz frequencies have been proposed [43,44,45,46]. As is well known, tunable unit-cell with a full 360° reflected phase coverage is crucial for realizing a high-performance reflectarray. A small reflected phase tunable range of the unit-cell often leads to deteriorative radiation performance, limiting the function of the whole reflectarray. However, phase tunable ranges of unit-cells in previously reported graphene-based reflectarrays or even metasurfaces [47] are essentially realized by tuning physical parameters of the graphene-based structures. Thus, due to the intrinsic resonant properties and finite losses (such asa damped oscillator) of the graphene-based material, only a narrow band phase tunable range of 300° has been presented [20,42,43,44], which restrict their applications in scenarios where high radiation performances are required. Especially, to the best of our knowledge, the CP graphene-based reflectarrays have also been seldom reported in previous literature, although there is a great demand on them at THz communications. One of the reason is that it is hard to achieve an excellent phase tunability by using graphene-based unit-cells. Therefore, designing a unit-cell with a wide-band tunable phase range of 360° is a meaningful and challenging work for the graphene-based reflectarray design.

In this article, we propose a graphene-based wide-band CP reflectarray operating in the THz regime. The proposed reflectarray consists of a THz CP source and several graphene-based unit-cells. Based on PB phase principle [48], the graphene-based unit-cell is designed to obtain a wide-band (1.4–1.7 THz) tunable phase range of 360°, which overcomes the restriction of intrinsic narrow band resonant in graphene. Therefore, the graphene-based unit-cell exhibits superior bandwidth and phase tunability to its previous counterparts. Based on the proposed graphene-based unit-cell, a focusing metasurface operating in 1.4–1.7 THz bands is demonstrated firstly, which exhibits prominent focusing effect. Then, according to the reversibility of the optical path, we realize a CP reflectarray by placing a wide-band CP THz source at the focal point of the metasurface. Numerical simulation demonstrates that this reflectarray can achieve a stable high gain up to 15 dBic and an axial ratio around 2.1 dB over the 1.4–1.7 THz bands. The good radiation performance of the proposed CP reflectarray, as demonstrated, underlines its suitability for the THz communication applications. 

## 2. PB Principle

**Figure 1:**
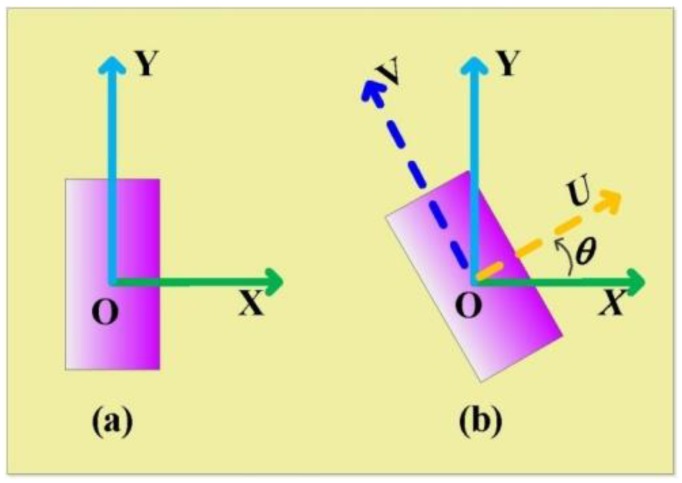
Illustration of PB phase unit-cell: (**a**) PB phase unit-cell in the *xy* coordinate; (**b**) PB phase unit-cell in the *uv* coordinate, which is transformed by an *θ* anticlockwise rotation of the original *xy* coordinate. Both coordinates have the same original point.

To have a clear physical insight of the proposed CP reflectarray, firstly, we have deduced the principle of PB phase, obtaining the necessary phase requirement of the unit cell. As shown in Figure 1, we apply a rectangle to represent a PB unit cell. In Figure 1a, a right-handed CP (RHCP) incident wave normally (along the −*z* direction) illuminates the surface, with an incident electric field expression

(1) $$E_{\mathrm{inc}}=(\vec{x}+j\vec{y})e^{j\vec{k}\vec{z}}$$

and the electric field of reflected wave will be

(2) $$E_{\mathrm{ref}}=(r_{x}\vec{x}e^{j\varphi_{x}}+jr_{y}\vec{y}e^{j\varphi_{y}})e^{-j\vec{k}\vec{z}}$$

where $\vec{k}$ denotes the wavenumber in free space. *r_x_* and *r_y_* represent the reflectivity of *x*-polarized and *y*-polarized waves, respectively. Similarly, *φ_x_* and *φ_y_* are the reflected phases of *x*-polarized and *y*-polarized waves, respectively.

As shown in Figure 1b, when the unit cell is anticlockwise rotated with an angle of *θ*, the relationship between the *uv* coordinate and the *xy* coordinate can be represented by

(3) $$\left\{ \begin{array}{l} \vec{x}=\vec{u}\cos\theta -\vec{v}\sin\theta \\ \vec{y}=\vec{u}\sin\theta +\vec{v}\cos\theta \end{array}\right.$$

Thus, in the *uvz* coordinate, we substitute Equation (3) into Equation (1), and the incident waves can be expressed as

(4) $$E_{\mathrm{inc}}=(\vec{u}+j\vec{v})e^{j\vec{k}\vec{z}}e^{j\theta }$$

and, corresponding reflected waves are

(5) $$E_{\mathrm{ref}}=(r_{u}\vec{u}e^{j\varphi_{u}}+jr_{v}\vec{v}e^{j\varphi_{v}})e^{-j\vec{k}\vec{z}}e^{j\theta }$$

From Figure 1, we can easily obtain that the reflectivity *r_u_* and reflected phase φ*_u_* in the *uvz* coordinate are equal to the reflectivity *r_x_* and reflected phase φ*_x_* in the *xyz* coordinate, respectively. In a similar way, the reflectivity *r_v_* and reflected phase φ*_v_* in the *uvz* coordinate are equal to the reflectivity *r_y_* and reflected phase φ*_y_* in the *xyz* coordinate, respectively. Therefore, *r_u_ = r_x_*, φ*_u_ =* φ*_x_*, *r_v_ = r_y_*, and φ*_v_ =* φ*_y_*. Then, Equation (5) can be expressed as

(6) $$E_{\mathrm{ref}}=\frac{1}{2}[(r_{x}\vec{x}-jr_{y}\vec{y})(e^{j\varphi_{x}}-e^{j\varphi_{y}})e^{j2\theta }+(r_{x}\vec{x}+jr_{y}\vec{y})(e^{j\varphi_{x}}-e^{j\varphi_{y}})]e^{-j\vec{k}\vec{z}}$$

From Equation (6), if *r_x_ = r_y_*, it is found that the reflected waves consist of two components, RHCP and left hand CP (LHCP), respectively.

(7) $$E_{\mathrm{ref}(\mathrm{RHCP})}=\frac{1}{2}r_{x}(\vec{x}-j\vec{y})(e^{j\varphi_{x}}-e^{j\varphi_{y}})e^{j2\theta }e^{-j\vec{k}\vec{z}}$$

(8) $$E_{\mathrm{ref}(\mathrm{LHCP})}=\frac{1}{2}r_{x}(\vec{x}+j\vec{y})(e^{j\varphi_{x}}+e^{j\varphi_{y}})e^{-j\vec{k}\vec{z}}$$

and when |φ*_x_* − φ*_y_*| = π, Equations (7) and (8) change to

(9) $$E_{\mathrm{ref}(\mathrm{RHCP})}=\frac{1}{2}r_{x}(\vec{x}-j\vec{y})e^{j\varphi_{x}}e^{-j\vec{k}\vec{z}}e^{j2\theta }$$

(10) $$E_{\mathrm{ref}(\mathrm{LHCP})}=0$$

Obviously, there is only an RHCP component inside the reflected wave, and the phase variation is 2*θ*, equal to two times of the rotation angle. Contrary to the metallic plate, the reflected waves of the PB unit-cell based metasurface only contain the co-polarized components. It is worth noting that the conditions of |φ*_x_* − φ*_y_*| ≈ π and *r_x_* ≈ *r_y_* must be fulfilled.

## 3. Graphene Based PB Unit-Cell

**Figure 2:**
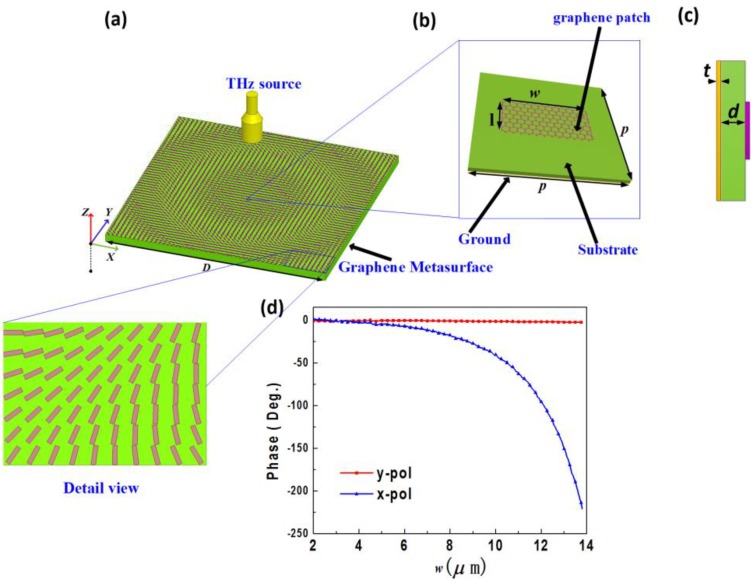
(**a**) Schematic of the proposed graphene-based reflectarray, which is composed of a focusing graphene metasurface and a CP THz source. (**b**) The PB unit-cell, which is composed of a rectangular graphene patch and a grounded quartz glass (SiO_2_) substrate. The side lengths of the rectangle are *w* and *l*, in *x* and *y* direction, respectively. *p* represents the side length of the unit-cell, which can be extended in both *x* and *y* direction, forming a 2-D metasurface. (**c**) Side view of the proposed PB unit-cell. Geometric parameters *t* and *d* denote the thickness of the ground plate and substrate, respectively. (**d**) Simulated reflected phases under the normal illumination of the *x-*and *y-*polarized plane waves. Fixing *l* = 3.2 µm, the variation range of parameter *w* is from 2 µm to 13 µm, at 1.52 THz.

Graphene can strongly interact with electromagnetic waves in THz regime through plasmonic resonance [23,24]. However, for practical applications, wave-graphene interactions have to be further improved. In this paper, a graphene-based PB phase unit-cell is proposed, as shown in Figure 2b. The structure of the unit-cell consists of a top layer rectangular graphene patch and a square grounded quartz glass (SiO_2_) substrate. When we extend these unit-cells periodically along both the *x* and *y* directions, a 2-D graphene-based metasurface can be obtained, as shown in Figure 2a. Incident terahertz waves can excite the plasmonic resonances of the graphene patches, and can be totally reflected by the bottom metallic ground. The top layer graphene-patches work as a partially reflecting mirror, and the bottom metallic ground operates as a fully reflecting mirror, respectively. Because of the rectangular shape of the graphene patch, the phases of the reflected waves can be independently manipulated by changing *w* or *l*, as shown in Figure 2d. The reason is that the *E_x_* component of the incident wave can only excite the plasmonic resonance in the *x* direction, and *E_y_* component of the incident wave can only excite the plasmonic resonance in the *y* direction [49]. Based on this characteristic, we can easily design a unit-cell fulfilling the conditions of |φ*_x_−*φ*_y_*| ≈ *π* and *r_x_* ≈ *r_y_*.

The modeling method of the graphene-based unit-cell is given in the following section. In the terahertz frequencies, the complex surface conductivity of graphene is determined by intraband transition. It can be approximated by a semi-classical Drude model [50]

(11) $$\sigma (\omega )=\frac{2e^{2}}{\pi \hbar^{2}}k_{B}T\ln[2\cosh(\frac{E_{f}}{2k_{B}T})]\frac{i}{\omega +i\tau^{-1}}$$

where *e* is the elementary charge, *k_B_* is the Boltzmann’s constant, ℏ is the reduced Plank’s constant, *T* is temperature, *τ* is the relaxation time, *ω* is the radian frequency, and *E_f_* is the Fermi energy. When *E_f_* is much larger than the thermal energy *k_B_ T*, the complex conductivity of graphene can be simplified to $\sigma (\omega )=\frac{e^{2}E_{f}}{\pi \hbar^{2}}\frac{i}{\omega +i\tau^{-1}}$. The electron relaxation time *τ* is the function of the carrier mobility *μ*, the Fermi energy *E_f_*, and Fermi velocity *v_f_*. It can be expressed as $\tau =\frac{E_{f}\mu }{ev_{f}{}^{2}}$, which implies that increasing *μ* will reduce the loss and enhance the efficiency of the device. The carrier mobility *μ* often has a variation range from ~1000 cm^2^/Vs to ~230,000 cm^2^/Vs with varied fabricated technologies [20]. In our simulation, according to the experimental results in Ref. [51], it is reasonable to assume that the Fermi energy *E_f_ =* 0.64 eV (corresponding to electron concentration of *n* = 3 × 10^13^ cm^−2^ in Ref. [51]), carrier mobility *μ* = 10,000 cm^2^/Vs, temperature *T =* 300 K and Fermi velocity *v_f_ =* 10^6^ m/s. 

The reflectivity and phases of the proposed unit-cell were full-wave simulated using Ansoft HFSS 2017 software (Ansoft, Pittsburgh, PA, USA). The unit-cell depicted in Figure 2b,c is modeled with a graphene patch on the top layer, then it was deposited on a square grounded SiO_2_ substrate. The SiO_2_ substrate has a relative permittivity of *ε_r_* = 3.75, and a loss tangent tan*δ* = 0.0184. The parameter *w* and *l* are the width and length of the graphene patch in the *x* and *y* directions, respectively, and *p* = 15 µm denotes unit-cell side-length which also equal to a periodicity to form the 2-D metasurface. Besides, *t =* 10 nm and *d =* 26 µm are the thickness of the metallic ground and quartz glass (SiO_2_) substrate, respectively. In our simulation, the master and slaver boundary condition was added to the unit-cell to modeling an infinite array. Meanwhile, Floquet port is placed at *z = 6d* and utilized to interact with the periodic unit-cell structure. In addition, the reflectivity and phases are obtained by the parametric sweep module of HFSS solver. The center frequency is set to 1.52 THz.

**Figure 3:**
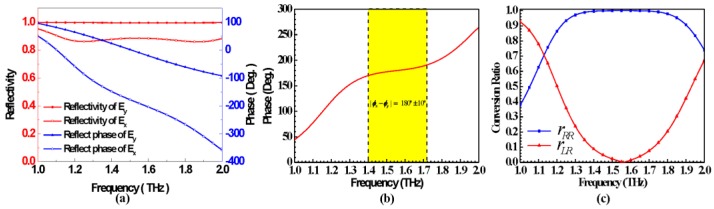
(**a**) Simulated reflection performance of the PB unit-cell under the normal illumination of *x-*and *y-*polarized plane waves. The red and blue curves represent the amplitude and phase of the reflected wave, respectively. Optimizing geometric parameters *w =* 13.39 µm and *l* = 3.2 µm are chosen in the simulation. (**b**) Simulated phase differences of the *x-*and *y-*polarized reflected waves. Yellow area in the figure demonstrates the frequency range of a nearly 180° phase difference. (**c**) Simulated co-polarized and cross-polarized conversion ratios under the normal RHCP incident plane wave. The blue and red curves represent the co-polarized conversion ratio and cross-polarized conversion ratio, respectively.

Such a PB unit cell can be looked as an anisotropic scatterer, which has a polarization dependent response. In normally incident case, we optimize the geometric parameters of the unit-cell, and choose the geometric parameters *w =* 13.39 μm, and *l =* 3.2 μm. Figure 3a displays the reflectivity and reflected phases of the *x-* and *y-*polarized waves, respectively. The reflectivity of *x-* and *y-*polarized waves are almost equal, with values near 1, indicating high efficiency. A nearly constant 180° reflected phase difference between the *x-* and *y-*polarized incident waves can be achieved in a wide frequency band (1.4–1.7 THz), realizing exactly 180° at 1.52 THz, as shown in Figure 3b.

According to the principle of PB phase, the proposed graphene-based unit-cell can realize co-polarized conversion, which can convert the illuminated CP wave into a co-polarized CP wave efficiently. Figure 3c gives the co-polarized and cross-polarized conversion ratios when there is a normally RHCP incident wave, verifying the co-polarized conversion. In 1.4–1.7 THz bands, high-efficiency co-polarized transformation has been achieved. Parameters *r_RR_* and *r_LR_* indicate the co-polarized conversion ratio and cross-polarized conversion ratio, respectively.

## 4. Graphene Metasurface for Focusing

After verifying the wide-band high efficiency co-polarized converting capability of the proposed PB unit-cell, we can compensate arbitrary phase in a range of 0°–360° by element rotating.

(12) $$\varphi (x,y)=\frac{2\pi }{\lambda_{0}}(\sqrt{x^{2}+y^{2}+f^{2}}-f)+\varphi_{0}$$

**Figure 4:**
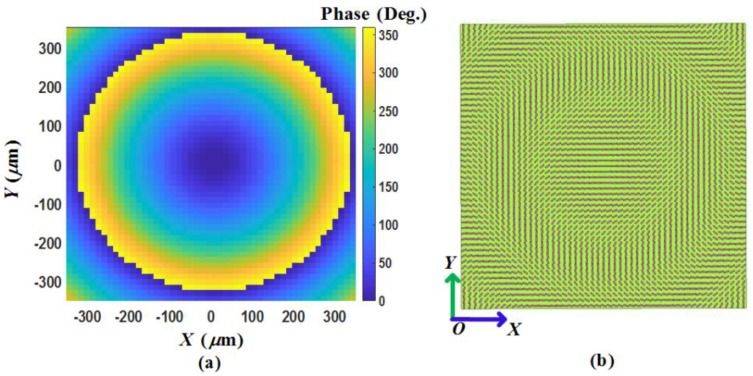
(**a**) The reflected phase distribution of the proposed focusing graphene metasurface with an area 714 µm × 714 µm. (**b**) The unit-cells distribution of the proposed focusing graphene metasurface with 51 × 51 unit-cells. Each unit-cell rotates a corresponding phase angle *θ = φ*/2.

**Figure 5:**
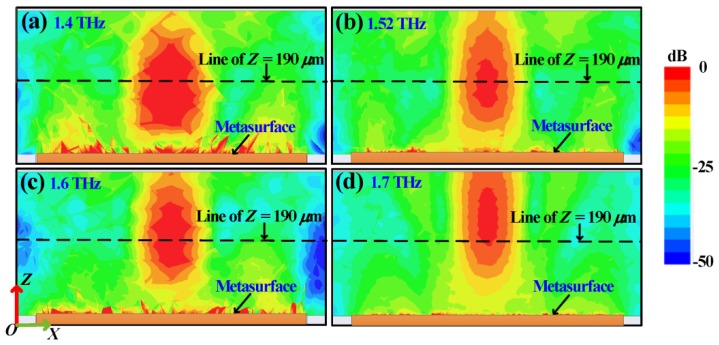
(**a**) Normalized energy distribution in *xoz* plane at 1.4 THz, the center *z* position of the focal point is less than 190 µm. (**b**) Normalized energy distribution in *xoz* plane at 1.52 THz, the center *z* position of the focal point is almost 190 µm. (**c**) Normalized energy distribution in *xoz* plane at 1.6 THz, the center *z* position of the focal point is a little larger than 190 µm. (**d**) Normalized energy distribution in *xoz* plane at 1.7 THz, the center *z* position of the focal point is quite larger than 190 µm.

To focus the reflected CP wave of a normally incident plane wave, a phase distribution must be fulfilled, where *f* represents focal length, *λ*_0_ represents wavelength in free space, *φ*_0_ represents initial phase at the original point. Following Equation (12), we design a focusing graphene metasurface with 51 × 51 unit-cells at 1.52 THz, and the focal length is set to 190 μm. The phase distribution is illustrated in Figure 4a, and corresponding unit-cell distribution is plotted in Figure 4b. In the numerical simulation, a normally RHCP incident wave is placed along the *−z* direction, obtaining energy distribution in the *xoz* plane at 1.4, 1.52, 1.6 and 1.7 THz, respectively, as depicted in Figure 5a–d. From these results, the prominent focusing effect can be observed, verifying our design. Furthermore, as the frequency increases, the focal length increases proportional, in accord with Equation (12). It is worth noting that a full 360° reflected phase range is quite important for generating such focusing metasurface. If narrower phase range is applied, as presented by previous studies [20,42,43], focusing performances will be degraded because of out of phase values in some positions of the metasurface.

## 5. High Gain CP Graphene-Based Reflectarray

**Figure 6:**
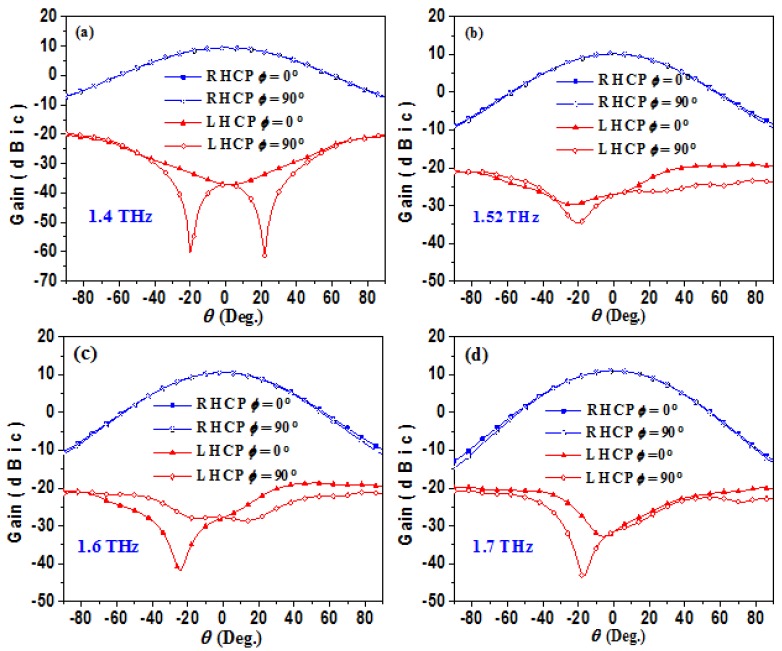
(**a**) Simulated 2-D radiation pattern of the feeding horn antenna at 1.4 THz. (**b**) The simulated 2-D radiation pattern of the feeding horn antenna at 1.52 THz. (**c**) The simulated 2-D radiation pattern of the feeding horn antenna at 1.6 THz. (**d**) The simulated 2-D radiation pattern of the feeding horn antenna at 1.7 THz. In all panels the gain of RHCP and LHCP components in *φ* = 0° plane and *φ =* 90° plane are demonstrated, respectively.

**Figure 7:**
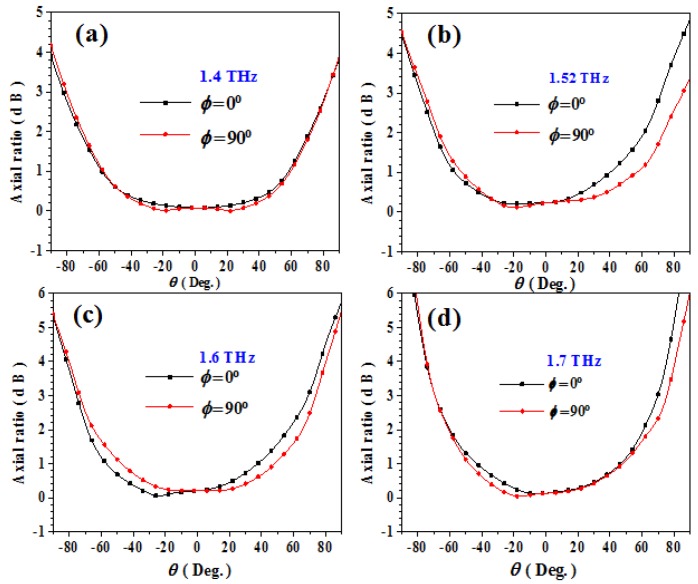
(**a**) Simulated axial ratio of the feeding horn antenna at 1.4 THz. (**b**) Simulated axial ratio of the feeding horn antenna at 1.52 THz. (**c**) Simulated axial ratio of the feeding horn antenna at 1.6 THz. (**d**) Simulated axial ratio of the feeding horn antenna at 1.7 THz. In all panels the axial ratios in *φ* = 0° plane and *φ* = 90° plane are demonstrated, respectively.

Previously, we have proposed a graphene metasurface which has prominent focusing capability in a wide-band of 1.4–1.7 THz. Then, according to the principle of optical path reversibility [52], we can place a wide-band CP THz source at the focal point of the metasurface. Thus, the incident spherical wave from the THz source can be converted to reflected plane wave with enhanced gain. In our simulation, we choose a THz CP horn antenna as the feeding sourceand optimize its radiation pattern (−10 dB beam width) for effective illumination. Simulated radiation patterns and axial ratios of the proposed feeding source at 1.4, 1.52, 1.6, and 1.7 THz are demonstrated in Figure 6 and Figure 7, respectively. Obviously, a stable −10 dB RHCP beam-width around 120° can be achieved, as shown in the figures. According to the calculated radiation angle, center feeding distance 190 μm is chosen, in keeping with the focal length *f* = 190 μm of the proposed metasurface.

**Figure 8:**
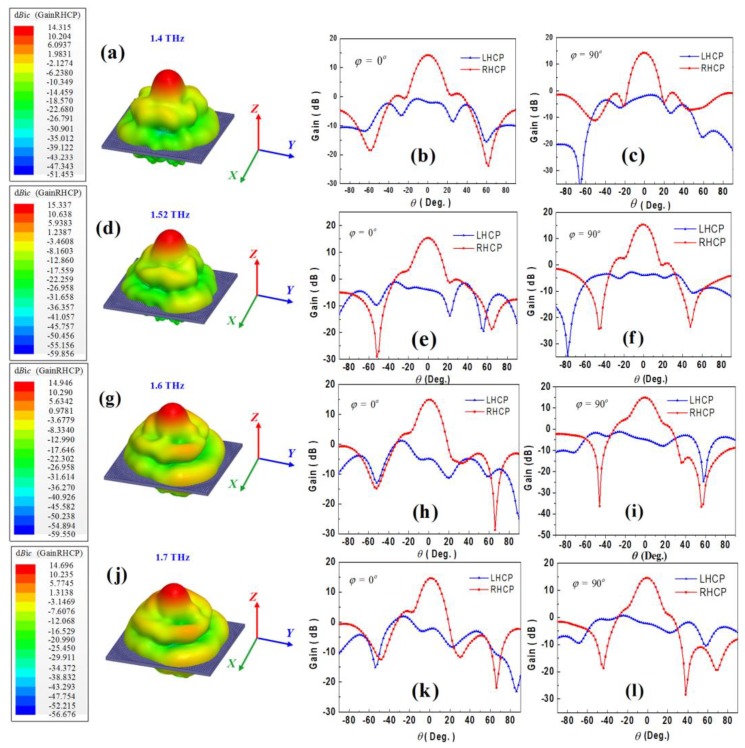
(**a**) 3-D radiation pattern of the proposed reflectarray at 1.4 THz. (**b**) 2-D radiation pattern of the proposed reflectarray at 1.4 THz in *ϕ* = 0° plane. (**c**) 2-D radiation pattern of the proposed reflectarray at 1.4 THz in *ϕ* = 90° plane. (**d**) 3-D radiation pattern of the proposed reflectarray at 1.52 THz. (**e**) 2-D radiation pattern of the proposed reflectarray at 1.52 THz in *ϕ* = 0° plane. (**f**) 2-D radiation pattern of the proposed reflectarray at 1.52 THz in *ϕ* = 90° plane. (**g**) 3-D radiation pattern of the proposed reflectarray at 1.6 THz. (**h**) 2-D radiation pattern of the proposed reflectarray at 1.6 THz in *ϕ* = 0° plane. (**i**) 2-D radiation pattern of the proposed reflectarray at 1.6 THz in *ϕ* = 90° plane. (**j**) 3-D radiation pattern of the proposed reflectarray at 1.7 THz. (**k**) 2-D radiation pattern of the proposed reflectarray at 1.7 THz in *ϕ* = 0° plane. (**l**) 2-D radiation pattern of the proposed reflectarray at 1.7 THz in *ϕ* = 90° plane.

**Figure 9:**
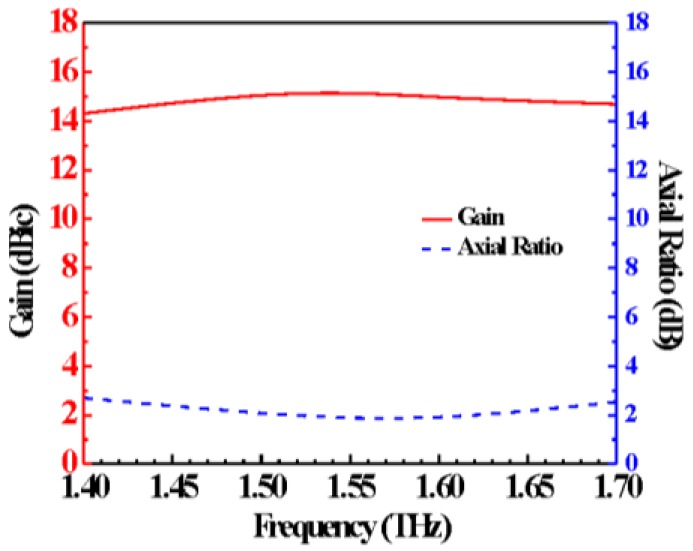
Simulated RHCP gain and axial ratio of the proposed reflectarray. The red and the blue curves represent the RHCP gain and axial ratio, respectively.

Figure 8a–l presents the radiation pattern of the reflectarray at 1.4, 1.52, 1.6 and 1.7 THz, respectively. It is shown that the enhanced gains produced by the proposed reflectarray are 14.32, 15.34, 14.95 and 14.70 dBic, at 1.4, 1.52, 1.6 and 1.7 THz, respectively. Besides, the cross-polarization levels are at least 15 dB lower than the co-polarization levels all across the 1.4–1.7 THz bands, demonstrating good CP properties. It is worth noting that the co-polarization radiation patterns have little asymmetric feature. Due to the center-feeding regime [53,54] of the proposed reflectarray, the CP feeding horn will inevitably interact with the reflected waves, causing distortion of the radiation pattern. Thus, we have to optimize the design parameters to maximally reduce the blockage effect of the feeding structure. Besides, we can alleviate such interaction by off-axial feeding regime. Furthermore, wide-band gain and axial ratio results are displayed in Figure 9, stable gain around 15 dBic and axial ratio around 2.1 dB can be achieved in the whole 1.4–1.7 THz bands, exhibiting both high gain and CP capability, simultaneously.

## 6. Conclusions

We have systematically investigated the spectral responses of graphene-based PB phase unit-cell and demonstrated a practical implementation of wide-band high gain CP reflectarray based on this configuration in the THz regime. Based on PB phase principle, an arbitrary reflected phase controlling can be realized, extremely extending the phase modulated ability of conventional graphene-based unit-cells that only have a phase controlling range of 300°. Finally, a focusing graphene-based metasurface and a reflectarray were designed, simulated and optimized. Simulation results exhibit excellent performances as theoretical expectations. The proposed reflectarray has a stable gain of 15 dBic and axial ratio of 2 dB over the 1.4–1.7 THz bands, demonstrating high gain and CP characteristics, simultaneously. This reflectarray may have a promising application in future THz communications. Meanwhile, for practical fabrication, we can design the graphene-based unit-cell which is consist of five layers, such as graphene layer, alumina layer, polysilicon layer, quartz glass layer, and ground. The polysilicon can be applied as an electrode. The Fermi energy is related to the conductivity of graphene, and can be dynamically tuned by varying the DC voltage (VDC) between the graphene and the polysilicon. Detailed techniques to fabricate these kinds of graphene reflectarray can be found in Reference [55], supporting the feasibility of our design. In addition, it is worth noting that the general technical procedure formulated herein facilitates further production of such graphene-based devices for various applications.
